# The Efficacy of Bezlotoxumab in the Prevention of Recurrent Clostridium difficile: A Systematic Review

**DOI:** 10.7759/cureus.27979

**Published:** 2022-08-13

**Authors:** Abhay Thandavaram, Aneeta Channar, Ansh Purohit, Bijay Shrestha, Deepkumar Patel, Hriday Shah, Kerollos Hanna, Harkirat Kaur, Mohammad S Alazzeh, Lubna Mohammed

**Affiliations:** 1 Internal Medicine, California Institute of Behavioral Neurosciences & Psychology, Fairfield, USA; 2 Family Medicine, California Institute of Behavioral Neurosciences & Psychology, Fairfield, USA; 3 Neurology, California Institute of Behavioral Neurosciences & Psychology, Fairfield, USA; 4 General Physician, California Institute of Behavioral Neurosciences & Psychology, Fairfield, USA; 5 Orthopedic Surgery, California Institute of Behavioral Neurosciences & Psychology, Fairfield, USA

**Keywords:** monoclonal antibody, clostridium difficile infection, clostridium difficile, bezlotoxumab, recurrent clostridium difficile infection

## Abstract

Clostridium difficile infection (CDI) is the most common nosocomial infection in hospitals. Despite the fact that CDI has treatment options, recurrence is common after the treatment, recurrence will occur in approximately 20%-35% of people initially affected, with 40%-60% of these having a second recurrence. Patients are more likely to have several recurrences after the second, which can lead to antibiotic overuse, and as a result, CDI-related health care expenses, hospitalizations, and mortality are on the rise. The first treatment to receive Food and Drug Administration (FDA) approval for the prevention of C. difficile recurrence is bezlotoxumab, a novel human monoclonal antibody against C. difficile toxin B. In the present systematic review, we assessed various studies from PubMed, PubMed Central (PMC), Google Scholar, and Science direct that evaluated the efficacy of bezlotoxumab in the prevention of recurrent C. difficile (rCDI), and we also briefly discussed the pathophysiology of C. difficile and the risk factors for recurrence of C. difficile. The major MODIFY trial has proven the efficacy, pooled analysis of MODIFY 1 AND 2 trials demonstrated the following results as compared to placebo (bezlotoxumab: 129/781 [16.5] placebo:206/773 [26.6] -10.0% [95% CI -14.0 to -6.0], p<0.0001) with number needed to treat (NNT) of 10. All other observational studies also showed a positive response with bezlotoxumab in the prevention of C. difficile. In conclusion, bezlotoxumab is a great option adjunctive with standard of care CDI antibiotics for the prevention of rCDI in high-risk adults.

## Introduction and background

Despite strong national and international concerns, the incidence of primary and recurrent C. difficile infection (PCDI and rCDI), respectively, has increased dramatically. Between 2000 and 2009, the prevalence of C. difficile in the United States more than doubled, and current estimates imply that C. difficile infects >500,000 patients yearly, resulting in over 14,000 deaths [1]. In 2011, C. difficile was responsible for about half a million infections and was linked to nearly 29,000 deaths. From 2012 to 2017, nucleic acid amplification test (NAAT) use was adjusted to 55% there was a decline in health-care-associated infections, and the anticipated total national burden of C. difficile infection (CDI) in the United States was reduced by an adjusted 24%. In 2017, community-associated infections did not decline and accounted for nearly half of all CDIs [2]. C. difficile has become the most common cause of health-care-associated infections in U.S. hospitals, with excess health-care expenses associated with CDI estimated at $4.8 billion for acute-care facilities alone in 2008 [3]. C. difficile, now Clostridioides difficile, is strongly linked to antibiotic use [4]. C. difficile is a gram-positive, toxin-producing anaerobic bacillus bacterium. The bacterium was difficult to culture when it was first described in newborns in 1935, subsequently named Bacillus difficilis. C difficile is ubiquitous and can be found in river water, soil, and meats. C difficile is also a spore-forming bacteria that can survive in harsh environments [5]. CDI is more common in the elderly and individuals with chronic medical conditions [6]. Following a primary bout of CDI, approximately 25% of individuals will develop a recurrent infection. After a first recurrence, the likelihood of a future recurrence rises to 40% [7]. The majority of recurrences are due to relapses of CDI with the original strain, rather than re-infection with a different strain [8]. Recurrent CDI infections put patients at risk for consequences such as toxic dilatation of the colon and septicemia, both of which have a high death rate [6]. C. difficile virulence factors include toxin A (TcdA) and toxin B (TcdB). TcdA and TcdB bind to and enter the colonic epithelium, resulting in the generation of proinflammatory chemokines and cytokines, neutrophil influx, tight junction rupture, fluid secretion, and epithelial cell death [4]. Toxin B is 10 times more potent than toxin A, thus strains that do not produce toxin A can be just as virulent as ones that produce both. NAP1/BI/027, a “hypervirulent” strain, produces a third toxin termed C. difficile binary toxin (CDT). The CDT toxin causes the gut wall to break down, facilitating adhesion [9].

CDI occurs most frequently during antibiotic usage and within the first month after treatment, but the risk can last up to 90 days. Proton pump inhibitor exposure has been detected in roughly 31% of community-acquired CDI patients, with no antibiotic exposure [8]. Metronidazole, vancomycin, and fidaxomicin are among the drugs used to treat primary illness, although they do little to prevent CDI recurrence. Fidaxomicin may lower the occurrence of rCDI when given early in patients with a non-NAP1/BI/027 strain; nevertheless, it did not appear to be more effective than oral vancomycin for the treatment of mild to moderate CDI. Fecal microbiota transplantation (FMT) has shown significant cure rates for rCDI, although there are concerns about regulatory issues and long-term safety. FMT's, widespread use, has been hindered by the lack of standardized ways of delivering fecal bacteria. In light of this, a human monoclonal antibody against toxin B (bezlotoxumab) was developed as an adjunct to antibiotic therapy to prevent rCDI [9]. The primary aim of this systematic review is to determine the efficacy of bezlotoxumab in the prevention of recurrent C. difficile.

## Review

Methods

Relevant studies were found by searching PubMed, PubMed Central(PMC), Google Scholar, and Science Direct. This review was carried out using the preferred reporting items for systematic review and meta-analysis (PRISMA) guidelines [10]. The following keywords were used for the search C. difficile, recurrent C. difficile, pseudomembranous colitis bezlotoxumab , Zinplava, and broadly neutralizing antibody. Additionally, a combination of the above regular keywords and the Medical Subject Headings (MeSH) strategy was used to identify relevant records from the PubMed databases.

Study Selection

Inclusion criteria were: published as randomized clinical trials (RCT) and observational cohort studies, Systematic Reviews, and Narrative reviews in the last 10 years (2012-2022), full free text. For Google Scholar, only the first 300 articles were reviewed because of the vast number of articles. There were no restrictions regarding age, sex, and duration of the study. We imposed no geographic or language restrictions. In observational studies, we included studies using bezlotoxumab to prevent recurrent CDI, with or without a comparator, in an adult population (18+ years) and a follow-up period of at least 90 days. Studies that failed to report clinical outcomes were excluded. All references were imported and managed in Zotero (Reference Manager). As shown in Table 1, the field search employed in the procedure was chosen based on keywords used in prior literature and Medical Subject Headings (Mesh), based on the database used.

**Table 1:** The method of conducting a bibliographic search in the databases using the appropriate filters PMC - PubMed central

Databases	Keywords	Search Strategy	Filters	Search Results
PubMed	bezlotoxumab OR Zinplava OR Broadly neutralizing antibody Clostridium difficile OR recurrent clostridium difficile OR Clostridium Infections OR pseudomembranous colitis	#1:Clostridium difficile OR recurrent clostridium difficile OR pseudomembranous colitis OR (( "Clostridium Infections/immunology"[Mesh] OR "Clostridium Infections/prevention and control"[Mesh] OR "Clostridium Infections/therapy"[Mesh])) OR ( "Clostridioides difficile/drug effects"[Mesh] OR "Clostridioides difficile/isolation and purification"[Mesh]) #2:bezlotoxumab OR Zinplava OR Broadly neutralizing antibody OR ("bezlotoxumab" [Supplementary Concept]) OR ("Antibodies, Monoclonal/isolation and purification"[Mesh] OR "Antibodies, Monoclonal/organization and administration"[Mesh] OR "Antibodies, Monoclonal/therapeutic use"[Mesh] OR "Antibodies, Monoclonal/toxicity"[Mesh]). #1 AND #2 - 225	Free full text, Meta-analysis, Systematic Review, Review articles, case reports, clinical study, observational studies, last 10 years, Humans	27
PMC	bezlotoxumab OR Zinplava OR Broadly neutralizing antibody Clostridium difficile OR recurrent clostridium difficile OR pseudomembranous colitis	#1:Clostridium difficile OR recurrent clostridium difficile OR pseudomembranous colitis OR (( "Clostridium Infections/immunology"[Mesh] OR "Clostridium Infections/prevention and control"[Mesh] OR "Clostridium Infections/therapy"[Mesh])) OR ( "Clostridioides difficile/drug effects"[Mesh] OR "Clostridioides difficile/isolation and purification"[Mesh]) #2:bezlotoxumab OR Zinplava OR Broadly neutralizing antibody OR ("bezlotoxumab" [Supplementary Concept]) OR ("Antibodies, Monoclonal/isolation and purification"[Mesh] OR "Antibodies, Monoclonal/organization and administration"[Mesh] OR "Antibodies, Monoclonal/therapeutic use"[Mesh] OR "Antibodies, Monoclonal/toxicity"[Mesh]). #1 AND #2 - 1144	Open access, last 5 years	731
Google scholar	Bezlotoxumab, clostridium difficile	Bezlotoxumab and clostridium difficile -1710	January 1, 2012 - May 24, 2022	1640
Science Direct	Bezlotoxumab, clostridium difficile	Bezlotoxumab and clostridium difficile-180	2012 - 2022, Review articles, research articles, conference abstracts, Case reports, Mini reviews, Only open archives.	39

Study Results and Bias Assessment

**Figure 1:**
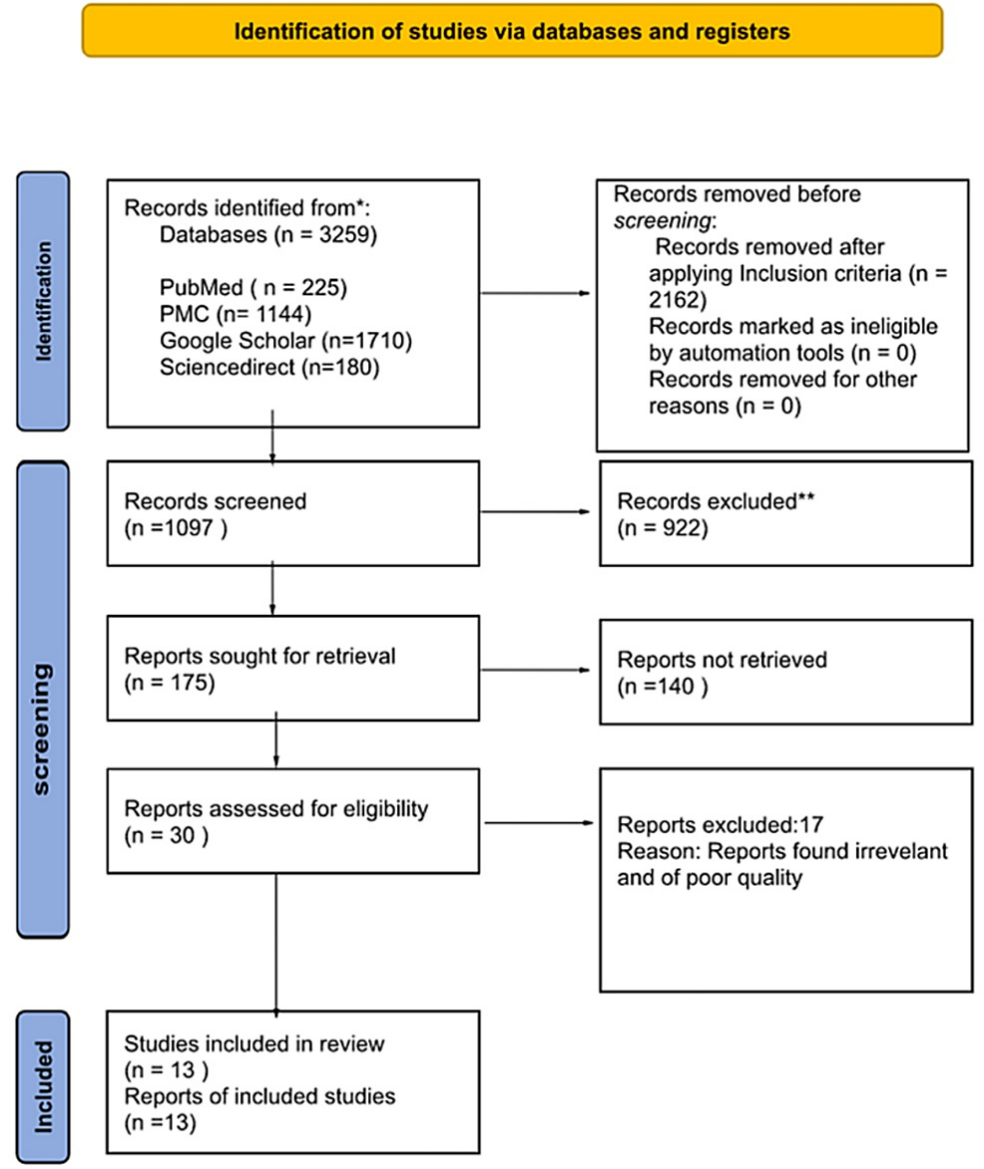
A flow diagram of the study selection PMC - PubMed Central

The initial search identified 3,259 publications. Of these, 2,162 articles were excluded after applying inclusion criteria. 1,097 articles were screened and an additional 922 were removed after screening titles and abstracts. Consequently, 175 papers were retrieved in full text. Of these, 30 articles were reviewed and 13 articles (One RCT, one systematic review, seven observational studies, and four reviews) were included in the review. Figure 1 shows a flow diagram depicting the screening process and study selection.

Risk of Bias

Randomized controlled trials were assessed with the Cochrane Collaboration’s risk of bias tool (CCRBT) [11]. For observational studies, the Newcastle Ottawa tool (NOS) [12] was used. For systematic review, Assessment of Multiple Systematic Reviews 2 (AMSTAR 2) checklist [13] was used and for Narrative reviews, Scale for the Assessment of Narrative Review Articles 2 (SANRA 2) checklist [14] was used. Each assessment tool required a score of at least 70% to be accepted (Table 2).

**Table 2:** Quality assessment of each study CCRBT - Cochrane Collaboration Risk of Bias Tool, NOS - Newcastle Ottawa Scale, AMSTAR 2 - Assessment of Multiple Systematic Reviews 2, SANRA 2 - Scale for the Assessment of Narrative Review Articles 2, RCTs - Randomized controlled trials, RoB - Risk of bias

Quality assessment tool	Type of study	Items & their characteristics	Total score	Accepted score (>70%)	Accepted studies
CCRBT [11]	RCT’s	Seven items: random sequence generation and allocation concealment (selection bias), selective outcome reporting (reporting bias), other sources of bias, blinding of participants and personnel (performance bias), blinding of outcome assessment (detection bias), and incomplete outcome data (attrition bias). Bias is assessed as LOW RISK, HIGH RISK, or UNCLEAR.	7	5	Wilcox et al. [15]
NOS [12]	Non-Randomised Control Trials and Observational studies	Eight items: (1) Representativeness of the exposed cohort (2) Selection of the non-exposed cohort (3) Ascertainment of exposure (4) Demonstration that an outcome of interest was not present at the start of study (5) Comparability of cohorts on the basis of the design or analysis* (6). Assessment of outcome (7) Was follow-up long enough for outcomes to occur (8) Adequacy of follow-up of cohorts Scoring was done by placing a point on each category. Scored as 0, 1, 2. * Maximum of two points are allotted in this category.	9	7	Herroro et al. [16], Askar et al. [17], Valerio et al. [18] Johnson et al. [19], Oksi et al. [20], Hengel et al. [21], Escudero-Sanchez et al. [22]
AMSTAR 2 [13]	Systematic reviews	Sixteen items: (1) Did the research questions and inclusion criteria for the review include the components of PICO? (2) Did the report of the review contain an explicit statement that the review methods were established prior to the conduct of the review, and did the report justify any significant deviations from the protocol? (3) Did the review authors explain their selection of the study designs for inclusion in the review? (4) Did the review authors use a comprehensive literature search strategy? (5) Did the review authors perform study selection in duplicate? (6) Did the review authors perform data extraction in duplicate? (7) Did the review authors provide a list of excluded studies and justify the exclusions? (8) Did the authors describe the included studies adequately? (9) Did the review authors use a satisfactory technique for assessing the risk of bias (RoB) in individual studies that were included in the review? (10) Did the review authors report on the sources of funding for the studies included in the review? (11) If meta-analysis was justified, did the review authors use appropriate methods for statistical combination of results? (12) If meta-analysis was performed, did the review authors assess the potential impact of RoB in individual studies on the results of the meta-analysis or other evidence synthesis? (13) Did the review authors account for RoB in individual studies when interpreting/ discussing the results of the review? (14) Did the review authors provide a satisfactory explanation for, and discussion of, any heterogeneity observed in the results of the review? (15) If they performed quantitative synthesis did the review authors carry out an adequate investigation of publication bias (small study bias) and discuss its likely impact on the results of the review? (16) Did the review authors report any potential sources of conflict of interest, including any funding they received for conducting the review? Scored as YES or NO. Partial Yes was considered as a point.	16	12	Alhifany et al. [23]
SANRA 2 [14]	Narrative review	Six items: justification of the article’s importance to the readership, statement of concrete aims or formulation of questions, description of the literature search, referencing, scientific reason, and appropriate presentation of data. Scored as 0, 1 or 2.	12	9	Giacobbe et al. [24], Alonso and Mahoney [7], Kelly and sangha [9], Kufel et al. [25]

Results

Table 3 describes the key characteristics of clinical trials, observational studies, and reviews.

**Table 3:** Main characteristics of the selected studies BEZ - Bezlotoxumab, SoC - Standard of Care, CDI - Clostridium difficile infection, rCDI - Recurrent clostridium difficile infection, AST - American society of transplantation, RCT - Randomized control trial, SOT - Solid organ transplant, HCT - Hematopoietic stem cell transplant, NNT - Number needed to treat, IBD - Inflammatory Bowel Disease, FMT- Fecal Microbiota transplant, PCR - Polymerase chain reaction, EIA - Enzyme immuno Assay, HO-HCFA - healthcare facility-associated, CO-HCFA - community-onset healthcare facility-associated, CA - community-associated, EOT - end of the study.

Reference	Study type	Patient Criteria	Primary outcome	Results	Conclusion
1. Wilcox et al. [15] (MODIFY 1 AND 2 TRIALS)	Double-blinded RCT	Eligibility Criteria: Included a diagnosis of CDI (diarrhea with positive toxigenic C. difficile test) and the age >= 18 Individuals taking 10 to 14 days of oral standard-of-care antibiotics (metronidazole, vancomycin, or fidaxomicin, as determined by the treating physician) for primary or recurrent C. difficile infection. In the trials, 2,580 (97%) of the 2655 participants were treated, and 2,559 (96%) were included in the modified intention-to-treat population.,2,174 people (85%) completed the 12-week trial. Randomization was stratified based on oral standard-of-care antibiotics and hospitalization status (inpatient or outpatient).	The proportion of participants with recurrent C. difficile infection (defined as a new episode of C. difficile infection after initial clinical cure of the baseline episode) during 12 weeks of follow-up in the modified intention-to-treat population.	(Pooled analysis) Bezlotoxumab: 129/781 (16.5) Placebo:206/773 (26.6) -10.0% (95% CI -14.0 to -6.0), p<0.0001 The number needed to treat to prevent one episode of recurrent C. difficile infection was 10; In subgroups of 65 years of age or older and those with previous C. difficile infection. The NNT was 6.	The outcomes of MODIFY I and MODIFY II, taken separately and together, reveal that bezlotoxumab was associated with a significantly reduced rate of recurrent infection than placebo among participants receiving standard-of-care antibiotic therapy for primary or recurrent C. difficile infection
2. Valerio et al. [18]	A retrospective observational study August 2018- september 2019	1. Patient that met criteria for bezlotoxumab financing in Spain. 2. Three or more risk factors for rCDI: age > 65 years, previous CDI episode, inability to quit antibiotics during CDI episode, immunosuppression (solid organ transplant(SOT), hematologic malignancy, neoplasia), infection by a hypervirulent strain such as the 027 ribotype, concomitant IBD, or low toxin B Ct values. 16 patients met bezlotoxumab selection criteria. Median age:69.5 years. 14 patients: Immunosuppressed. Nine patients: Previous CDI (with a total of 15 episodes/recurrences treated with metronidazole (1), vancomycin (7), extended duration vancomycin (2), or fidaxomicin (5)).	Recurrence of clostridium difficile in the first (10-90) days after recovery	As two individuals died from unrelated causes, no CDI cure could be demonstrated. Of the remaining 14 patients, 11 did not recur during a Three-month follow-up period. This resulted in a 21.4% recurrence rate.	For patients who refuse FMT, those who may have contraindications to it, or at hospitals where it is not available, bezlotoxumab could be a feasible alternative.
3.Johnson et al. [19]	A retrospective cohort study at the University of Colorado Hospital (UCH), a 700-bed academic tertiary care institution. Between January 2015 and November 2019	The following criteria were included: (1) age 18–89 years; (2) SOT or HCT history; (3) the identification of CDI via positive C. difficile polymerase chain reaction (PCR) results and the onset of clinically significant diarrhea as per AST guidelines; (4) the administration of SoC CDI antibiotics (oral vancomycin [VAN], fidaxomicin [FDX], or metronidazole [MTZ]); and (5) a follow-up visit recorded 90 days after the end of therapy. 649 patients that were screened, 39 in BEZ and 56 in SoC, were found to be eligible for the trial. The average age was 53 years.	Incidence of rCDI at 90 days after completion of CDI antibiotics.	In unadjusted analysis, there was no difference between BEZ and SoC participants in the primary outcome of 90-day rCDI (16% vs 29%, P =.13). In a multivariable study of 90-day rCDI incidence in the general population, BEZ was linked to a 72% decreased risk of rCDI when compared to those who did not take BEZ (odds ratio, 0.28 [95% CI, .08–.91]; P =.03)	Overall, findings herein suggest that high-risk SOT/ HCT recipients may derive benefit from BEZ.
4. Oksi et al [20]	A retrospective observational study In 2017, the efficacy and safety of BEZ were retrospectively assessed in an intent-to-treat scenario at all five university hospitals in Finland (Helsinki, Oulu, kuopio, Tampere, and Turku). Method of testing: all hospitals used the polymerase chain reaction (PCR) approach	In April–December 2017, the first 46 patients in Finland to get BEZ were enrolled. Patients who were in the hospital as well as those who had already been discharged were allowed for analysis. Mean age of all patients was 66 years (range 15–97 years). Due to underlying comorbidities or immunosuppressive medication, 28 (or 61%) of the 46 patients were immunocompromised. Eight risk variables for CDI recurrence were present in 78% of patients: 16 (35%) had five or more risk factors, 20 (43%) had three or more, and 10 (22%) had just one or two. 37 of the 46 patients with SOC received vancomycin, nine received metronidazole, seven received fidaxomicin, and two received tigecycline.	Recurrent Clostridium difficile infection	In all, 32 (73%) of 44 patients did not experience rCDI in the three months following BEZ infusion. Two patients were excluded from the category of "remaining free of rCDI" because they died before three months had passed following the BEZ infusion.	BEZ infusion as an adjuvant treatment to SOC was effective in preventing rCDI in 73% of patients, and it was also effective in immunocompromised patients, with a performance of 71%. In cases with severe CDI, 63% of cases remained rCDI-free over the next three months.
5. Hengel et al. [21]	A retrospective, multicenter cohort study. Patients who received bezlotoxumab between April 2017 and December 2018 were retrospectively evaluated at 34 infusion facilities across the United States.	Bezlotoxumab was given in combination with SoC to 200 patients from 34 US physician infusion facilities to prevent rCDI. The median age (range) was 70 years (21–98) Prior to receiving bezlotoxumab, 73 patients (36.5%) were hospitalized for a mean of 5+/- 4 days within four weeks of their current CDI episode, the majority (n = 67) due to CDI. At the start of the study, 27 patients (13.5%) had primary CDI, 50 (25.0%) had one recurrence, 62 (31.0%) had two recurrences, and 61 (30.5%) had three CDI recurrences. Risk factor distribution: Age >65 years (n = 134, 67.0%), compromised immunity (n = 84, 42.0%), current CDI with severe presentation (n = 56, 28.0%), and one CDI episode in the last 6 months (n = 154, 77.0%) were among the rCDI risk factors. Overall, 158 patients (79.0%) had two of the four risk variables, while 65 patients (32.5%) had three. Oral antibiotic distribution: Oral SoC antibiotics administered in combination with bezlotoxumab were vancomycin fixed dosage (n = 76, 38.0%), vancomycin tapered regimen (n = 61, 30.5%), fidaxomicin (n = 60, 30.0%), and metronidazole (n = 3, 1.5%).	Recurrence of Clostridium difficile	In 195 of 200 patients, recurrence was assessed, with 31 individuals (15.9%) suffering rCDI within 90 days. All the patients experienced recurring diarrhea, necessitating medical attention in 23 of them (22 PCR, one EIA), and eight patients also had positive C. difficile stool tests.	84.1% of patients experienced successful prevention of rCDI. Following the administration of a single dose of bezlotoxumab in conjunction with SoC treatment in US outpatient infusion centers,
6.Escudero-Sánchez et al. [22]	Between July 2018 and July 2019 in 13 Spanish hospitals, a retrospective, multicenter cohort analysis of patients receiving bezlotoxumab treatment for CDI was carried out.	In the database, there were 91 consecutive patients from 13 different centers. The Patients were on average 71 years old, with 46 (50.5%) of them being men Bezlotoxumab was given to 39 (42.9%) patients during the initial CDI episode, 28 (30.8%) during the initial recurrence, and 24 (26.4%) during the second or subsequent recurrences. According to current definitions, patients were categorized as either healthcare facility-onset, healthcare facility-associated (HO-HCFA) in 39 (42.9%) patients, community-onset, healthcare facility-associated (CO-HCFA) in 35 (38.5%) patients, community-associated (CA) in 11 (12.1%) patients, or indeterminate in 6 patients (6.6%).	the rate of rCDI during the 12 weeks after the end of antimicrobial treatment for CDI.	13 out of 91 (14.3%) patients acquired rCDI after a median follow-up time Of 74 (49–81) days after the completion of treatment and 84 (81–89) days after the infusion of bezlotoxumab.	Despite the presence of a significantly more vulnerable, at-risk group, the rCDI rate was equivalent to that found in the MODIFY trials. The outcomes in the sample were unaffected by the type of Anti-C. Difficile medication regimen. Regardless of age, severity, or comorbidities, bezlotoxumab has shown favorable effects.
7. Herroro et al. [16]	A longitudinal, retrospective study of a cohort of patients treated with bezlotoxumab in the tertiary hospital in spain. 2 August 2018 and 31 March 2021	A total of 52 patients were enrolled in The study. A single infusion of bezlotoxumab (10 mg/kg) was given to each patient. The median age was 73.5 years, with 32 (61.5%) women 42.9% of patients received bezlotoxumab for the initial CDI episode, 22 (30.8%) for the initial recurrence and 14 (26.4%) for the second or subsequent recurrences. During the recurrence, 32 patients (61.54%) received vancomycin at the standard dose, while 16 patients (30.77%) used vancomycin tapering and four (7.69%) used fidaxomicin.	The proportion of clinical cure within 12 weeks was the key variable	Within 12 weeks of receiving bezlotoxumab, there were nine (18.4%) recurrences. Six patients died during their inpatient stay, and three more died during the 12-week follow-up period, therefore were not included in the recurrence ratio calculation. The recurrence ratio was 20.9% after three months of bezlotoxumab treatment, which is identical to what was seen in pivotal clinical studies (16.5%)	The recurrence ratio was 20.9% after 3 months of bezlotoxumab treatment, which is identical to what was seen in pivotal clinical studies (16.5%). Recurrences were shown to be more common in the subgroup of patients with severe CDI.
8. Askar et al. [17]	An Observational study in tertiary care center	A total of 29 patients were referred for BEZ Those who received BEZ were compared to those who did not receive BEZ in a cohort of patients who were referred for BEZ (standard of care, SOC). BEZ was given to 14 people (48%). Patients with high risk for recurrent infection (history of solid organ transplant (SOT) or hematopoietic stem cell (HCT) transplantation, active malignancy, chronic steroid (prednisone equivalent 20 mg/day), and failed fecal microbiota transplant (FMT).	rCDI after 100 days of BEZ infusion or at the end of the study (EOT).	With an NNT of 7, the rCDI at 100 days was 14.3% BEZ vs. 28.6% SOC (P = 0.3654). The BEZ group took longer to reach rCDI than the SOC group (49 vs. 27 days)	Early results with BEZ in a high-risk, primarily immunocompromised group are encouraging. The NNT for rCDI prevention was 7. Larger cost–benefit analyses in immunocompromised and transplanted patients are needed.
9.Alhifany et al. [23]	A Systematic review Evaluating the effectiveness and safety of fecal microbiota transplantation in reducing the risk of recurrent Clostridium difficile infections in comparison to bezlotoxumab.	Eligibility Criteria: After a brief course of SATs, RCTs that assessed the effectiveness and safety of FMT and bezlotoxumab in treating CDI. SAT such as vancomycin, Metronidazole, or fidaxomicin were used. Both published and unpublished, were eligible for inclusion. 1)If they had included patients 18 years or older diagnosed with RCDI 2)Reported the resolution rate of CDI as the efficacy outcome	The resolution of CDI-related diarrhea without relapse for at Least 60 days after therapy has ended. Adverse outcomes.	There is no statistically significant difference between FMT and bezlotoxumab (OR 1.53, 95% CrI 0.39 to 5.16). Despite this, FMT had the highest SUCRA probability (63.6%). Furthermore, FMT outperformed SAT in terms of CDI resolution (OR 2.98, 95% CrI 1.13 to 7.53). Bezlotoxumab, on the other hand, revealed no statistical difference in CDI resolution when compared to SAT (OR 1.93, 95% CrI 0.84 to 4.91)	FMT infusions, whether single or multiple, were equally effective in resolving RCDI as bezlotoxumab infusions, but with a higher rate of non-serious diarrhea. More research is needed to determine the efficacy and safety of utilizing FMT as a monotherapy for CDI, as well as the potential attenuating effect of short-course antibiotics administered before FMT and the clinical consequences of numerous bezlotoxumab infusions.
10. Giacobbe et al. [24]	Narrative Review	In February 2019, the authors were given separate topics to research using inductive PubMed searches: (1) CDI pathophysiology; (2) bezlotoxumab chemistry and mechanism of action; (3) bezlotoxumab pharmacology; (4) efficacy of bezlotoxumab in phase 3 randomized controlled trials (RCTs); (5) bezlotoxumab in observational studies; and (6) safety of bezlotoxumab in clinical studies They were then instructed to write different drafts on their allocated study topic. The drafts were eventually combined into a comprehensive manuscript that was reviewed and approved by all the authors.			CDI and rCDI continue to be linked to decreased patient quality of life and higher healthcare costs. Bezlotoxumab has shown to be effective in lowering the burden of rCDI, presenting clinicians with an essential new method for achieving long-term cure in CDI patients.
11. Alonso and Mahoney et al. [7]	Narrative review	The phrases "bezlotoxumab," "Zinplava," "MK-6072," "MDX1388," "Clostridium difficile or Clostridioides difficile and monoclonal antibody," and "Clostridium difficile or Clostridioides difficile and antitoxin" were used in a PubMed search from 1946 to August 2018. All English-language data were examined, with a focus on therapy and safety data in humans and animals. Additional reviews using Embase, the Cochrane database library, Web of Science, clinicaltrials.gov, and abstracts from significant infectious disease and gastroenterology conference proceedings were conducted.			Bezlotoxumab is a fully-humanized monoclonal antibody directed against C. difficile toxin B that is used to prevent rCDI in patients who are at risk. Its novel mechanism of action, apparent lack of effect on the fecal microbiome, and favorable safety profile make it a promising adjunctive therapy for rCDI prevention. Real-world clinical data from the next several years should offer light on the drug's efficacy in the highest-risk CDI populations, as well as how it compares to other innovative preventative medicines
12.Kufel et al. [25]	Narrative review				The first-in-class, FDA-approved drug to promote passive immunity for the prevention of CDI recurrence is bezlotoxumab, a completely humanized monoclonal antibody that binds to and neutralizes C. difficile toxin B. Bezlotoxumab was well tolerated and effective in clinical trials for reducing CDI recurrence when compared to placebo. To guide cost-effective use, pharmacoeconomic evaluations are required. Although phase 4 clinical experience will disclose much of the significance of bezlotoxumab, it is a welcome addition to the CDI management arsenal, where therapeutic alternatives are limited.
13.Kelly and Sangha et al. [9]	Narrative review				The treatment of recurrent CDI appears to have a bright future. Participants with three risk factors experienced the greatest reduction in CDI recurrence with bezlotoxumab, while those with one or two risk factors also benefited considerably. Bezlotoxumab should be evaluated in the treatment of high-risk individuals over 65 years old with several risk factors to avoid CDI recurrence. Patients who are taking antibiotics at the same time, have inflammatory bowel disease, or are not responding to FMT may benefit from bezlotoxumab medication.

Discussion

The following sections discuss the pathophysiology of C. difficile, risk factors for recurrent CDI and the role of bezlotoxumab in the prevention of rCDI.

Pathophysiology of C. difficile

C. difficile's potential to cause enteritis is determined by two host characteristics: colonization resistance and immunological response to C. difficile. The indigenous flora of the large intestine, which consists of around 4,000 bacterial species and is collectively known as the fecal microbiome, protects it from invasive diseases. By competing for vital nutrients and attachment sites to the gut wall, these microorganisms give colonization resistance against pathogenic species. Antibiotics disrupt the barrier microbiota and reduce colonization resistance, creating an environment for gut infections to colonize. Antibiotic reduction of the Bacteroides and Firmicutes phyla appears to be particularly relevant in the pathogenesis of C. difficile [26]. C. difficile pathophysiology is mostly based on the effects of toxin A and toxin B. The toxins are encoded by the tcdA and tcdB genes, which are located within a chromosomally integrated DNA sequence known as the pathogenicity locus or PaLoc. Three more genes are found in the PaLoc: (1) tcdR, which codes for an alternative RNA polymerase sigma factor that regulates the expression of tcdA and tcdB. (2) tcdE, which encodes a putative holin-like protein required for both toxins' extracellular release; and (3) tcdC, which inhibits TcdA and TcdB production. PaLoc can be horizontally transferred from non-pathogenic strains lacking tcdA and tcdB, transforming them into pathogenic strains to producers. In theory, while a “healthy” gut microbiota converts primary bile acids into secondary bile acids (which inhibit C. difficile germination), a disrupted microbiota, deficient in primary bile acid converters, may enable C. difficile germination and overgrowth following broad-spectrum antibiotic therapy [24].

Toxins are delivered into the cytoplasm of the host cell in seven steps: (1) toxin binding to a receptor on the surface of the host cell; (2) Internalization of the toxin via receptor-mediated endocytosis; (3) acidification of endosome; (4) formation of the pore; (5) release of glucosyltransferase domain (GTD) from the endosome to the host cell cytoplasm; (6) inactivation of Rho GTPases by glucosylation; and (7) downstream consequences on the host cell, such as cytotoxic and cytopathic effects brought on by the toxin. Cytopathic effects: Blocking Rho-dependent signaling disrupts the actin cytoskeleton and tight and adherent junctions, resulting in the loss of cell-cell connections and increased epithelial permeability, all of which are possible reasons for diarrhea. Poor cell adhesion leads to apoptosis and cell loss. Epithelial cell renewal is limited, and cell proliferation is hindered as a result of the suppression of both cell cycle progression and actin-dependent cytokinesis. Type I (apoptosis) and type III (necrosis) programmed cell death can result from the cytotoxic effects of TcdA and TcdB. TcdA can induce apoptosis predominantly via activating caspase-8 and cytochrome c/caspase-9, which has drawn attention to the role of TcdA glucosyltransferase activity in this process And is dependent on Rho protein monoglucosylation.TcdB has the ability to activate a variety of apoptotic pathways in host cells. In addition to glucosylation, TcdB activities also result in cytotoxicity, mostly through caspase-dependent (activation of caspase-3) and caspase-independent mechanisms (i.e., Bcl-2-dependent mechanism) apoptotic mechanisms. TcdB has also been shown to cause apoptosis through the involvement of mitochondrial ATP-dependent potassium channels. This process is linked to an increase in cytosolic calcium concentration and hyperpolarization of the mitochondrial membrane, a state that is likely to influence commitment to cell death. The cytotoxic effects are also linked to the activation of the inflammasome by glycosylated RhoA, which is thought to be the cause of C. difficile-induced inflammation and colitis [27].

Risk Factors for Recurrent C. difficile

CDI was defined as diarrhea (≥Three unformed bowel movements [types 5 to 7 on the Bristol stool scale] in 24 hours) with a stool test result that was positive for toxigenic C. difficile [15]. Recurrences are linked to a weakened immune response to C. difficile toxins and/or changes in the microbiota in the colon [28]. Advanced Age: The most frequently reported risk factor for rCDI is advanced age. The reason for the recurrence in elderly people is unclear, decreased immune response to CDI and increased comorbidity may play a role [29]. Antibiotic use: The alteration of the gut microbiota by antibiotics impacts nutrient sources in at least two ways. Antibiotics reduce competition for limited resources and open up previously unoccupied ecological niches by lowering the diversity of the intestinal microbiota. Second, bacterial cell lysis provides carbon sources that can be consumed by the remaining community. The altered intestinal microbiota by antibiotics also influences bile acid composition in the colon, thereby promoting the growth of C. difficile [29,30]. Gastric acid suppression: These agents may reduce stomach acidity, weakening defenses against C. difficile and increasing the risk of CDI. The rate of rCDI in patients with stomach acid suppression was higher than in patients without gastric acid suppression, according to a recent meta-analysis that included 16 observational studies with 7,703 CDI patients (22.1% vs 17.3%: OR, 1.52; 95% CI, 1.20 to 1.94; p 0.001) [29,31]. Hypervirulent strains: The strain NAP1/BI/027 is resistant to fluoroquinolones, which has been linked to geographically distributed CDI epidemics. Patients with strain NAP1/BI/027 exhibited a greater recurrence rate than patients with non-hypervirulent strains (27.4% vs 16.6%, p = 0.002) in a clinical trial of 719 CDI patients [29,32]. Other important risk factors: Host genetics, compromised immune system, Chronic renal failure, Prior CDI episode Severity of CDI episode and Severe Primary CDI, and Prolonged Hospital stay [24,29].

Role of Bezlotoxumab in the Prevention of Recurrent C. difficile

Bezlotoxumab is a fully humanized ImmunoglobulinG1 (IgG1) monoclonal antibody that only binds to C. difficile toxin B. Binding to toxin B neutralizes the toxin and prevents damage to mammalian colonic cells. Currently, bezlotoxumab is approved for the prevention of rCDI in adult patients at high risk for rCDI. The product must be administered during the active CDI antibacterial treatment and is available as 1000 mg/40 mL single-dose vials. Reconstituted vials should be diluted in 0.9% sodium chloride or 5% dextrose to a final concentration between 1 and 10 mg/mL. The recommended dosage is based on the patient's body weight, with 10 mg/kg intravenously over 60 min in a single administration, up to treatment day 14 [2,3].

MODIFY I and MODIFY II were two separate but largely identical trials by Wilcox et al. that was designed to see if bezlotoxumab alone or in conjunction with actoxumab (Monoclonal antibody against C. difficile toxin A) could prevent rCDI. MODIFY I also included an actoxumab alone arm, which was dropped early after being linked to significantly higher rates of sepsis-related death and a lack of efficacy when compared to the bezlotoxumab and actoxumab arm. Randomization was stratified based on oral standard-of-care antibiotics and hospitalization status (inpatient or outpatient). The primary endpoint was the proportion of participants with rCDI (defined as a new episode of CDI after the initial clinical cure of the baseline episode) during 12 weeks of follow-up in the modified intention-to-treat population. The initial clinical cure was defined as no loose stools for two consecutive days after completing standard-of-care (SoC) antibiotic therapy for ≤16 days. A secondary endpoint, also called global cure or sustained clinical response, was the sustained cure rate, which meant initial clinical cure as defined above and no recurrence of CDI through 12 weeks [15]. 

In the trials, 2,580 (97%) of the 2,655 participants were treated, and 2,559 (96%) were included in the modified intention-to-treat population. In the modified intention-to-treat population, 2,174 people (85%) completed the 12-week trial. In both trials, the percentage of participants with recurrent infection in the modified intention-to-treat population was significantly lower in the bezlotoxumab group than in the placebo group (MODIFY I: 17% [67 of 386] vs. 28% [109 of 395]; adjusted difference, −10.1 percentage points; 95% CI, −15.9 to −4.3; p < 0.001; MODIFY II: 16% [62 of 395] vs. 26% [97 of 378]; adjusted difference, −9.9 percentage points; 95% CI, −15.5 to −4.3; p < 0.001 [15]. In the actoxumab-bezlotoxumab group, recurrence was considerably lower than in the placebo group (MODIFY I: 16% [61 of 383] vs. 28% [109 of 395]; adjusted difference, −11.6 percentage points; 95% CI, −17.4 to −5.9; MODIFY II: 15% [58 of 390] vs. 26% [97 of 378]; adjusted difference, −10.7 percentage points; 95% CI, −16.4 to −5.1; both p < 0.001). The majority of recurrences (71%) occurred within four weeks of the study infusion. Differences in the risk of recurrent infection between the bezlotoxumab and placebo regimens were visible as early as two weeks after infusion and lasted until week 12. The number needed to treat (NNT) to prevent one episode of rCDI was 10. In subgroups of 65 years of age or older and those with previous CDI the NNT was 6. The outcomes of MODIFY I and MODIFY II, taken separately and together, reveal that bezlotoxumab was associated with a significantly reduced rate of recurrent infection than placebo among participants receiving SoC antibiotic therapy for primary or rCDI [4,15]. The most common adverse events reported during the first four weeks after infusion were nausea, vomiting, abdominal pain, diarrhea, fatigue, pyrexia, urinary tract infection, and headache [15].

In the observational study by Valerio et al., bezlotoxumab was only financed for patients with three or more risk factors. Recurrent CDI (r-CDI) was defined as CDI symptoms and positive stool samples that occurred in the first 10 to 90 days after recovery from a previous CDI episode. Out of 16 patients in the study, two patients received fidaxomicin (because of vancomycin allergy) and 14 patients received vancomycin. As two patients died, out of 14 patients there were only three recurrences with a recurrence rate of 21.4%. Limitations of this study include there was no comparison group, and it was done in a small group of patients in an institution [18].

In the observational study by Johnson et al., the effectiveness of bezlotoxumab was assessed only in the transplant patients as they’re at high risk for rCDIn and its sequela of graft loss and mortality. 39 in the bezlotoxumab (BEZ) group and 56 in the SoC group. During BEZ administration, one patient suffered nausea and vomiting, prompting her to stop taking medication. In unadjusted analysis, there was no difference between BEZ and SoC participants in the primary outcome of 90-day rCDI (16% vs 29%, p = 0.13). In a multivariable study of 90-day rCDI incidence in the general population, BEZ was linked to a 72% decreased risk of rCDI when compared to those who did not take BEZ (odds ratio, 0.28 [95% CI, .08-0.91]; p = 0.03). The number of previous CDI episodes was likewise linked to a higher risk of rCDI. The most common CDI treatment (86%) was PO Vancomycin (VAN), which was consistent across cohorts (p = 0.99;). BEZ patients were more likely to receive Fidaxomicin (FDX) (34% vs 11%, p = 0.01), while SoC recipients were more likely to use combination treatments (mainly PO VAN + IV Metronidazole (MTZ), 13% vs 41%, p = 0.01). Less than 50% of patients in this study have more than four risk factors and Immunocompromised patient populations have been underrepresented in the pivotal trials, accounting for about 20% of the MODIFY I/II. Patients in the MODIFY studies were given BEZ a median of three days after starting CDI-directed drugs. Due to the average hospital stay being quite long, reimbursement issues with inpatient administration, insurance prior permission restrictions, and scheduling issues for outpatient infusion, patients in this study received BEZ at a median of 25 days after CDI treatment initiation. Limitations of the study include: the study's primary and secondary outcomes were under powered, and these findings should be taken as exploratory, Patients who received prophylaxis during courses of broad-spectrum antibiotics and tapering regimens with recurrent CDI had longer durations of CDI therapy when receiving BEZ [19].

In the observational study by Oksi et al., out of 46 patients, seventeen patients were outpatients (eight of whom were awaiting FMT), while 29/46 patients were in the hospital at the time of BEZ infusion. Due to background comorbidity or immunosuppressive treatment, 28 (61%)/46 patients were immunocompromised. After treatment with BEZ, 20 (71%) / 28 immunocompromised patients did not have a rCDI. 19 (42%) of the 45 patients (one patient was unknown) were treated with at least one antibacterial antibiotic (other than anti-C. difficile). In 37 of the 46 patients with SoC, vancomycin was used partly or entirely, metronidazole in nine, fidaxomicin in seven, and tigecycline in two. In 73% of patients, adjunctive treatment to SoC was effective in preventing rCDI, and the performance remained at 71% effective even among immunocompromised individuals. In cases with severe CDI, 63% of cases remained rCDI-free over the next three months. BEZ had no significant side effects, but one patient reported shocking feelings after the infusion, and another patient developed a fever the next day after the infusion. Within a three-month follow-up period, 42% of participants in this study received concurrent antibiotics. The best results for preventing rCDI were achieved when BEZ was given in a stable setting, such as right before discharge from the hospital or after all antibiotic treatments, including SoC antibiotics, had been stopped (as was the case with those receiving BEZ when waiting for fecal microbiota transplantation [FMT]). In this condition, the intestinal flora has more time to recuperate, which helps with healing. Limitations include use of solely toxin gene PCR to detect CDI. C. difficile is likely to colonize patients for some time following the SOC. However, in the hospitals where the study was conducted, there is a high bar for using the test-it is not permitted to be used unless there is a genuine clinical suspicion of CDI or its return. Furthermore, the use of BEZ was always supervised by a specialist in infectious disease [20].

In the observational study by Hengel et al., of 200 patients, 27 had primary CDI and 50 had one recurrence, 62 had two recurrences and 61 had three or more than three recurrences. Age 65 years (n = 134, 67.0%), impaired immunity (n = 84, 42.0%), current CDI with severe presentation (n = 56, 28.0%), and one CDI episode in the last six months (n = 154, 77.0%) were among the rCDI risk factors. Overall, 158 patients (79.0%) had two of these four risk factors, while 65 patients (32.5%) had three. Of the additional risk factors included, 86 patients (43.0%) were on gastric acid suppressants, 58 (29.0%) were on non-CDI antimicrobials within four weeks prior to current CDI, 35 (17.5%) had the chronic renal illness, 23 (11.5%) had previously failed FMT, 18 (9.0%) had inflammatory bowel disease, and 10 (5.0%) had a history of CHF. PCR was used for 153 patients and Enzyme Immunoassay was used for 47 patients for C. Difficile confirmation. Oral SoC antibiotics administered in combination with bezlotoxumab were vancomycin fixed dosage (n = 76, 38.0%), vancomycin taper regimen (n = 61, 30.5%), fidaxomicin (n = 60, 30.0%), and metronidazole (n = 3, 1.5%). Following bezlotoxumab, 17 (55%) of rCDI patients were treated with SoC antibiotics, 12 (39%) with SoC plus FMT, and two (6%) with FMT alone. Hospitalization was required in 11 of the 31 individuals with rCDI, three of whom had severe illness. Recurrence rates with vancomycin fixed dosage, vancomycin taper, and fidaxomicin were 13.7%, 18.3%, and 15.2%, respectively, depending on the SoC regimen employed. A CDI recurrence was seen in one out of every three patients (33%) in the metronidazole group. Those in the rCDI group experienced significantly higher CDI recurrences than patients in the nonrecurrent group (80.6% vs 57.9%; p = 0.017). The recurrence rate is 15.9 comparable to 16.5% of the Pivotal MODIFY trial and rCDI was clinically defined as recurrence of diarrhea for ≥two days leading to medical intervention regardless of the availability of a positive C. difficile stool test. Because confirming diagnostic tests were not done consistently in this investigation, a higher proportion of diarrhea recurrences could have been diagnosed as rCDI. The recurrence rate of patients with previously failed FMT was equivalent to that of those without FMT. Since both cohorts were excluded from the MODIFY trials, this is the first instance of bezlotoxumab being used in patients who had previously failed FMT or had received a tapering vancomycin SoC regimen. Limitations of this include: Due to the lack of C. difficile ribotyping, it was difficult to link recurrence to the CDI strain. Documentation of Gastric acid suppressant therapy could be underestimated, as it was patient-reported [21].

In the observational study by Escudero Sanchez et al. 91 patients were included in the study Along with the five established risk factors of MODIFY trial (age over 65, previous CDI episode, immunosuppression, infection owing to a hypervirulent strain, and severe episode), other important variables included for comparison are 1. Renal impairment 2. Positive direct toxin detection in the feces 3.Fidaxomicin treatment. After a median follow-up period of 84 days after infusion of bezlotoxumab 13/91 (14.3%) met with rCDI comparable to the pivotal MODIFY trial. Recurrence was defined as the reappearance of disease symptoms after symptom resolution from the previous episode, as well as a positive test demonstrating the presence of toxigenic C. difficile in the stool during follow-up. The rate of rCDI was higher in patients who had two or more CDI episodes (25% vs 10.4%; P=0.09) Although the strain ribotype was only determined in 48 patients in this study, the recurrence was found to be greater in patients with the 027 ribotype (4 out of 10; 40%). No difference in recurrence rate was observed based on anti C.difficile drug given, age, the severity of the episode, and the Microbiological technique used. No adverse events were reported in the study. The use of fidaxomicin for CDI treatment was the sole factor that could have favored a reduced recurrence rate (somewhat more frequently observed in our cohort). However, because only 13% of patients received this treatment, we cannot consider the influence of fidaxomicin as a meaningful factor in this study. Because 13 patients died before the 12-week follow-up period ended, it's possible that the recurrence rate we reported was overestimated. However, these patients' median follow-up until death was nearly identical to our cohort's median time until rCDI. Even if these individuals were not included in the analysis, the recurrence rate (16.7%) would be comparable to the MODIFY trials. Limitations include: This is a retrospective, multicenter cohort, therefore there's a chance of heterogeneity and data loss. Some cases of rCDI may have been missed, since definition of recurrence needed microbiological evidence of toxigenic C. difficile [22].

In the observational study by Herroro et al. ,out of 52 patients during the recurrence, bezlotoxumab was administered to 42.9% of patients during the initial CDI episode, 22 (30.8%) during the first recurrence, and 14 (26.4%) during subsequent recurrences. 32 patients (61.54%) received vancomycin at the standard dose, while 16 patients (30.77%) used vancomycin tapering and four (7.69%) used fidaxomicin. Within 12 weeks of receiving bezlotoxumab, there were nine (18.4%) recurrences. As nine patients died during the study, they were not included in the study and recurrence ratio is reported as 20.9% [16].

In the observational study by Askar et al., bezlotoxumab was evaluated in patients with high risk for recurrent infection (history of a solid organ (SOT) or hematopoietic stem cell (HCT) transplantation, active malignancy, chronic steroid (prednisone equivalent 20 mg/day), and failed fecal microbiota transplant (FMT). Among 29 patients, 14 received bezlotoxumab. With an NNT of 7, the rCDI at 100 days was 14.3% BEZ vs. 28.6% SOC (p = 0.3654). The BEZ group took longer to reach rCDI than the SOC group (49 vs. 27 days) [17].

In the systematic review by Alhifany et al., in the absence of head-to-head RCTs, a Bayesian network meta-analysis was conducted to compare the efficacy and safety of bezlotoxumab versus FMT in lowering the risk of rCDI in hospitalized patients. FMT and bezlotoxumab efficacy and safety in resolving CDI after a short course of SAT such as vancomycin, metronidazole, or fidaxomicin, patients aged 18 and up who were diagnosed with rCDI and reported the CDI resolution rate as the efficacy outcome were eligible for inclusion in both published and unpublished RCTs. The binary outcomes were expressed as odds ratio (OR) and 95% credible interval (95% CrI) for rCDI resolution rate and OR with 95% CrI for adverse events. The surface under the cumulative ranking curve (SUCRA) method was used to calculate treatment ranking probabilities. A sensitivity analysis was also performed to exclude studies and/or patients who received non-Food and Drug Administration(non-FDA)-approved mAB. Four open-label RCTs involving 139 patients compared FMT to vancomycin alone in patients with an initial episode of CDI or rCDI and were followed for at least 70 days after the treatments ended. The efficacy of mABs was investigated in three double-blind, placebo-controlled RCTs, including two multicenter phase II studies and one multinational, multicenter phase III study. The preliminary analysis comparing the resolution of CDI after one FMT infusion versus any mAB regimen discovered there was no statistically significant difference between FMT and bezlotoxumab (OR 1.53, 95% CrI 0.39 to 5.16). Nonetheless, FMT had the highest SUCRA probability (63.6%). Furthermore, FMT outperformed SAT in CDI resolution (OR 2.98, 95% CrI 1.13 to 7.53). Bezlotoxumab, on the other hand, showed no statistically significant difference in CDI resolution when compared to SAT (OR 1.93, 95% CrI 0.84 to 4.91). Limitations of the review include: The RCT’s included are of variable quality, as more than 50% of the studies did not report blinding of the participants. Furthermore, the RCTs investigating FMT differed in terms of design, donor selection, FMT preparation, follow-up time, lag time between feces collection and infusion, and lag time between antibiotic discontinuation and FMT infusion; whereas mABs were infused either during or immediately after antibiotic discontinuation. The number of previous recurrences was not reported in any of the included RCTs. Furthermore, due to the early termination of the majority of the included RCTs and inconsistent reporting of adverse events, safety outcomes were limited [23].

In the narrative reviews by Giacobbe et al. and Alonso and Mahoney, MODIFY trials and Post hoc analysis of MODIFY trials were reviewed. In one of the post hoc analysis, Participants in the MODIFY trials who had sustained clinical cure at 12 weeks had no rCDI after another nine months of follow-up (0/69, 0%) versus 2/65 (3%) and 1/34 (3%) in the bezlotoxumab plus actoxumab and placebo groups, respectively [2]. Zeng and colleagues used whole-genome sequencing to distinguish recurrences from new infections. Recurrences due to relapse of infection by the same ribotype of the index CDI episode (198/259 evaluable patients, 76%) were distinguished from recurrences due to a different ribotype (50/259 evaluable patients, 19%) [33]. The researchers noted that patients receiving bezlotoxumab had a lower cumulative incidence of relapses (as measured by a competing risk model) than patients who did not receive bezlotoxumab (actoxumab or placebo) [33]. Decreased CDI-related hospital readmissions in patients who are at risk of rCDI [5.1% (27/530) vs. 11.2% (58/520), with a difference of 6.1%, 95%] CI - 9.5 to - 2.8 was observed in post hoc analysis by prabhu et al. [34]. Bezlotoxumab resulted in a gain of 0.12 QALYs (Quality Adjusted Life Years) compared to placebo, and appeared cost-effective in terms of the prevention of rCDI in the entire study population, with an incremental cost-effectiveness ratio of US$19 824/QALY gained in the post hoc analysis by prabhu et al. [35]. Endogenous antibodies against toxin B were protective compared to antibodies against toxin A, which is consistent with the results of bezlotoxumab as compared to actoxumab [36]. In terms of bezlotoxumab administration timing, efficacy in preventing rCDI was unaffected by the time of administration in relation to the onset of antibiotic treatment (i.e. 0-2, 3-4, and greater than five days after onset) [2,3,37]. The proportion of patients developing rCDI in 382 MODIFY I/II participants with cancer was lower in the bezlotoxumab arms (26/146, 17.8%) than in the placebo arms (42/138, 30.4%), with an absolute difference of - 12.6%, 95% CI - 22.5 to - 2.7% [38]. An exploratory study looked into whether human genetic variants can affect how bezlotoxumab works in patients included in the MODIFY trials. In individuals treated with bezlotoxumab, the single nucleotide polymorphism rs2516513 and the human leukocyte antigen alleles HLA-DRB1*07:01 and HLADQA1*02:01, which are found in the extended major histocompatibility complex on chromosome 6, were linked to a lower incidence of rCDI. The same was not observed in patients who were given a placebo [2,3,39].

Limitations

There are some limitations to our analysis, the minimal number of publications from only four databases were included and grey literature and other databases are excluded, the evaluation of solely free full-text papers, and the small sample sizes and lack of comparison group in the majority of the observational studies. The criteria for patient selection, the definition of recurrence, the timing of antibiotic administration, and the method of testing are varied between studies. Because of the significant level of variability among the studies included in our analysis, our results should be interpreted with caution.

## Conclusions

In summary, this systematic review shows that bezlotoxumab, a recently approved novel agent by FDA, has proven to be effective in the prevention of rCDI in high-risk individuals. It has also shown to be effective in decreasing hospital readmission and improved quality of life from Post hoc studies. Its negligible impact on the fecal microbiome, and ability to use in outpatient infusion centers, make it a promising supplementary therapy for rCDI prophylaxis. To assess the efficacy of FMT versus bezlotoxumab in preventing recurrence, head-to-head randomized clinical trials will be needed. In light of few therapeutic options available for the prevention of rCDI, bezlotoxumab is a great option with a good safety profile.
